# Low frequency of enterohemorrhagic, enteroinvasive and diffusely adherent *Escherichia coli* in children under 5 years in rural Mozambique: a case-control study

**DOI:** 10.1186/s12879-020-05380-1

**Published:** 2020-09-07

**Authors:** Marcelino Garrine, Glória Matambisso, Nélio Nobela, Delfino Vubil, Sérgio Massora, Sozinho Acácio, Tacilta Nhampossa, Pedro Alonso, Inácio Mandomando

**Affiliations:** 1 Centro de Investigação em Saúde de Manhiça (CISM), Maputo, Mozambique; 2 Global Health and Tropical Medicine (GHTM), Instituto de Higiene e Medicina Tropical (IHMT), Universidade Nova de Lisboa (UNL), Lisbon, Portugal; 3 Instituto Nacional de Saúde (INS), Ministério da Saúde, Maputo, Mozambique; 4 ISGlobal, Hospital Clínic-Universitat de Barcelona, Barcelona, Spain

**Keywords:** Diarrhea, Diarrheagenic *Escherichia coli* pathotypes, PCR, Children

## Abstract

**Background:**

Diarrheagenic *Escherichia coli* (DEC) are among the leading pathogens associated with endemic diarrhea in low income countries. Yet, few epidemiological studies have focused the contribution of enterohemorrhagic *E. coli* (EHEC), enteroinvasive *E. coli* (EIEC) and diffusely adherent *E. coli* (DAEC)*.*

**Methods:**

We assessed the contribution of EHEC, EIEC and DAEC isolated from stool samples from a case-control study conducted in children aged < 5 years in Southern Mozambique between December 2007 and November 2012. The isolates were screened by conventional PCR targeting *stx1* and *stx2* (EHEC), *ial* and *ipaH* (EIEC), and *daaE* (DAEC) genes.

**Results:**

We analyzed 297 samples from cases with less-severe diarrhea (LSD) matched to 297 controls, and 89 samples from cases with moderate-to-severe diarrhea (MSD) matched to 222 controls, collected between November 3, 2011 and November 2, 2012. DEC were more common among LSD cases (2.7%, [8/297] of cases vs. 1.3% [4/297] of controls; *p* = 0.243]) than in MSD cases (0%, [0/89] of cases vs. 0.4%, [1/222] of controls; *p* = 1.000). Detailed analysis revealed low frequency of EHEC, DAEC or EIEC and no association with diarrhea in all age strata. Although the low frequency, EIEC was predominant in LSD cases aged 24–59 months (4.1% for cases vs. 0% for controls), followed by DAEC in similar frequency for cases and controls in infants (1.9%) and lastly EHEC from one control. Analysis of a subset of samples from previous period (December 10, 2007 and October 31, 2011) showed high frequency of DEC in controls compared to MSD cases (16.2%, [25/154] vs. 11.9%, [14/118], *p* = 0.383, respectively). Among these, DAEC predominated, being detected in 7.7% of cases vs. 17.6% of controls aged 24–59 months, followed by EIEC in 7.7% of cases vs. 5.9% of controls for the same age category, although no association was observed. EHEC was detected in one sample from cases and two from controls.

**Conclusions:**

Our data suggests that although EHEC, DAEC and EIEC are less frequent in endemic diarrhea in rural Mozambique, attention should be given to their transmission dynamics (e.g. the role on sporadic or epidemic diarrhea) considering that the role of asymptomatic individuals as source of dissemination remains unknown.

## Background

Diarrheal illness is one of the major cause of morbidity and mortality among children less than 5 years of age, accounting for 526,000 of the 5.942 millions of deaths globally [1]. Studies on diarrheal diseases from South America and Africa have reported prevalence of bacterial pathogens ranging between 27 and 56%, with diarrheagenic *Escherichia coli* (DEC) being one of the most frequent identified pathogen in children with diarrhea [2–5]. DEC have been traditionally classified in six groups based on their clinical, epidemiological and virulence traits: enterotoxigenic *E. coli* (ETEC), enteropathogenic *E. coli* (EPEC), enteroinvasive *E. coli* (EIEC), enteroaggregative *E. coli* (EAEC), diffusely adherent *E. coli* (DAEC) and enterohemorrhagic *E. coli* (EHEC) [6–8]. Among these, ETEC, EPEC and EAEC have consistently been associated with endemic diarrheal, while EHEC is often associated with outbreaks [8]. In contrast, the epidemiology and the role of DAEC on diarrheal remain unclear in most developing countries [9, 10]; while EIEC has been reported sporadically with low frequency in Africa [4, 11].

In Mozambique, despite the trend of diarrheal decline over the years (1997–2011), it remains among the major causes of morbidity and mortality in children aged less than 5 years [12], particularly in Manhiça district [13]. Studies reporting the occurrence of DEC in Mozambique remain scarce, and often focus on molecular screening of the three most common pathotypes (EAEC, ETEC and EPEC) [5, 11, 14–17] rather than all DEC. As part of the Global Enteric Multicenter Study (GEMS) we have previously demonstrated that ETEC, producing stable toxin (ST-ETEC) was the only *E. coli* pathotype associated to moderate-to-severe diarrhea (MSD) [16] and less-severe diarrhea (LSD) [17] in toddlers (12–23 months) in Mozambique. However, GEMS did not assess the potential role of less common pathotypes (EIEC, EHEC and DAEC). Therefore, in this study, we investigated the contribution of those pathotypes on diarrheal etiology in children aged < 5 years enrolled in the GEMS in Manhiça, southern Mozambique between December 10, 2007 and November 2, 2012.

## Methodology

### Study site description

The study was conducted by the Manhiça Health Research Centre (Centro de Investigação em Saúde de Manhiça - CISM), in Manhiça district, a rural area in Maputo Province, southern Mozambique. The district is located approximately 80 km north of Maputo city with two distinct seasons, rainy and hot season from November to April and dry and cool season from May to October, as detailed elsewhere [18, 19]. The CISM has been running a continuous Demographic Surveillance System (DSS) for vital events and migrations since 1996 [18], currently covering the entire Manhiça district with an estimated population of 183,000 inhabitants in 43,000 households with two regular visits a year to update demographic events (migration, births, deaths, etc.).

### Study design

We analyzed a subset of *E. coli* isolates collected under the GEMS protocol conducted between two distinct periods, that aimed to quantify the burden, etiology and sequelae of diarrhea in sub-Saharan Africa and South Asia [16]. The first period (December 10, 2007 and October 31, 2011) comprised only the enrolment of children with MSD and respective controls without diarrhea [16], while the second one (November 3, 2011 and November 2, 2012) comprised two parallel case-control studies, one assessing MSD and the other LSD; each with their respective controls [17]. In the GEMS study, children less than 5 years with diarrhea (≥3 loose stools within the previous 24 h) belonging to the catchment area under DSS who sought care at the sentinel health centers (SHCs) were assessed for study criteria and enrolled in LSD or MSD group. MSD was defined as a new (onset after ≥7 diarrhea-free days) and acute (onset within the previous 7 days) diarrheal episode presenting one of the following criteria: sunken eyes, loss of skin turgor, intravenous hydration administered or prescribed, dysentery, or hospitalization [20]. LSD was defined as a new acute diarrhea case seen at SHCs that did not meet the definition of MSD. For each case, 1–3 community based controls (children free of diarrhea in the last 7 days) were enrolled matched by age stratum (infants [0–11 months], toddlers [12–23 months] and young children [24–59 months]), neighborhood and gender as described elsewhere [20]. At enrolment, each participant provided fresh stool that was placed in cold chain and transport media according to the protocol. If antibiotics were to be administered to participants with diarrhea before stool was produced, we obtained two rectal swabs for bacterial culture pending passage of the whole stool for the remaining assays [20].

### Specimen processing for pathogen detection

In GEMS protocol, the stool samples from cases and controls were assessed for numerous enteropathogens (bacterial, protozoal and viral agents) using microbiological and molecular methods [17, 21]. For *E. coli* isolation, stool samples were plated onto MacConkey (MAC) agar and incubated at 37 °C for 18–24 h. Afterward, putative lactose-fermenting bacterial colonies resembling *E. coli* were picked and tested using Motility Indole Ornithine medium. Up to 3 lactose and indole positive *E. coli* colonies per sample were selected and stored at − 80 °C in single colony for further analysis [21]. In GEMS protocol, the *E. coli* colonies were analyzed by multiplex PCR that targeted only for ETEC, EAEC and EPEC [21]. Therefore, in this investigation, the *E. coli* colonies from GEMS were screened by conventional PCR targeting additional pathotypes of DEC as EHEC (*stx1* and *stx2*), DAEC (*daaE*) and EIEC (*ial* and *ipaH*). Briefly, the three putative *E. coli* colonies isolated from stools in GEMS study were retrieved onto MAC and incubated at 37 °C for 18–24 h. Afterward, the *E. coli* colonies from same stool were pooled, the DNA extracted [21] and analyzed by multiplex PCRs using primers previously designed for detection of *ial* [22], *ipaH* [23], *daaE* [24], *stx1* and *stx2* genes [21]. We performed two multiplex PCRs; the first one detected genes *ial, ipaH* and *daaE* and the second one the genes *stx1* and *stx2*. For the first multiplex, 3 μl of DNA template was added to the PCR mix containing 12.5 μl of PCR Mix 2X (Qiagen), 5 μl of Q-solution 10X (Qiagen), 0.5 μl of 10 μM of each primer and 1.5 μl of RNase-free water to a final volume of 25 μl. The second multiplex contained: 12.5 μl of PCR Mix 2X (Qiagen), 5 μl of Q-solution 10X (Qiagen), 0.2 μl of 25 μM of each primer, 3.7 μl of RNase-free water and 3 μl of DNA template to a final volume of 25 μl. A single cycling protocol was applied for both multiplex, with following parameters: preheating at 95 °C for 15 min; and 35 cycles of denaturation at 94 °C for 30 s, annealing at 57 °C for 90 s, elongation at 72 °C for 90 s; with final extension at 72 °C for 10 min in an Eppendorf Mastercycler Gradient thermal cycler (Eppendorf, Hamburg, Germany). The amplification products were separated through a 2% agarose gel stained with ethidium bromide. The 1-kb plusA 100-bp DNA ladder (Bio-Rad) was used as a molecular size marker in gel.

### Statistical analysis

The statistical analyses were performed using STATA version 14.1 (StataCorp LP, College Station, Texas, USA). We assessed associations of MSD and LSD with each pathotype (EHEC, EIEC e DAEC) using Chi-squared test or Fisher’s exact test, as appropriate. We deemed *p-*value of 0.05 or lower to be statistically significant.

## Results

### Diarrheagenic *Escherichia coli* isolated between November 3, 2011 and November 2, 2012

We analyzed 297 stools samples from cases with LSD matched to 297 controls (children without diarrhea), and 89 stool samples from cases with MSD matched to 222 controls, collected between November 3, 2011 and November 2, 2012. Among the cases with LSD, 120 children aged 12–23 months, 104 were infants, and 73 were young children (Table 1). In contrast, the MSD study showed that, diarrhea was most frequent in infants (42 cases matched to 99 controls), followed in children aged 12–23 months (29 cases matched to 79 controls) and lastly in young children (18 cases matched to 44 controls). We also found that diarrhea was more frequently reported in male children than in female children, both in LSD (54.2%, 161/297) and in the MSD study (64.2%, 57/89).

**Table 1:** Diarrheagenic *E. coli* frequency in children with LSD enrolled between November, 2011 and November, 2012

Pathotype	0–11 months			12–23 months			24–59 months		
	Cases ***n*** = 104 (%)	Controls ***n*** = 104 (%)	P	Cases ***n*** = 120 (%)	Controls ***n*** = 120 (%)	P	Cases ***n*** = 73 (%)	Controls ***n*** = 73 (%)	P
EIEC	1 (1)	0	1	1 (0.8)	1 (0.8)	1	3 (4.1)	0	0.245
DAEC	2 (1.9)	2 (1.9)	1	1 (0.8)	0	1	0	0	–
EHEC	0	1 (1)	1	0	0	–	0	0	–

DEC were more common among LSD cases (2.7%, [8/297] of cases vs. 1.3% [4/297] of controls; *p* = 0.243]) than in MSD cases (0%, [0/89] of cases vs. 0.4%, [1/222] of controls; *p* = 1.000). Detailed analysis of DEC circulation for the above period showed a low frequency of EHEC, EIEC and DAEC in all age strata with no significant association with neither LSD nor MSD. Although the low frequency of pathotypes among cases in the LSD group, EIEC was predominant in children aged 24–59 months (4.1% [3/73] for cases vs. 0% for controls). This was followed by DAEC in children from 0–11  months (1.9% [2/104] for cases vs. 1.9% [2/104] for controls) and lastly EHEC being isolated in one sample from control (Table 1). Among the MSD group, DAEC was only detected in one sample from control (0–11 months), while EIEC and EHEC were absent.

### Diarrheagenic *Escherichia coli* isolated between December 10, 2007 and October 31, 2011

The analysis of a subset of *E. coli* (118 from cases with MSD and from 154 matched controls) isolated between December 10, 2007 to October 31, 2011 revealed remarkable variation of pathotypes EHEC, EIEC and DAEC frequencies when compared to the period between November 3, 2011 and November 2, 2012. We observed high frequency of DEC in controls compared to MSD cases (16.2%, [25/154] vs. 11.9%, [14/118], *p* = 0.383, respectively) between December 10, 2007 to October 31, 2011. Among these, DAEC was the most common pathotype, being detected in a similar proportion in cases (8.4%; 6/71) and controls (8.4%; 8/95) during infancy. Nonetheless, DAEC frequency was higher in controls aged 12–23 months (0% for cases vs. 7.1% for controls) and 24–59 months (7.7% for cases vs. 17.6% for controls). In contrast, EIEC was found more frequent in cases than in controls for all age strata, although no association was observed (Table 2). EHEC was only found in three samples; one sample from case (infant) and one from control (infant) and in one sample from a control aged 24–59 months.

**Table 2:** Prevalence of DEC in children with MSD enrolled between December, 2007 and October, 2011

Pathotype	0–11 months		12–23 months		24–59 months	
	Cases ***n*** = 71 (%)	Controls ***n*** = 95 (%)	Cases ***n*** = 34 (%)	Controls ***n*** = 42 (%)	Cases ***n*** = 13 (%)	Controls ***n*** = 17 (%)
EIEC	4 (7.4)	7 (5.6)	2 (5.9)	2 (4.8)	1 (7.7)	1 (5.9)
DAEC	6 (8.4)	8 (8.4)	0	3 (7.1)	1 (7.7)	3 (17.6)
EHEC	1 (1.4)	1 (1)	0	0	0	1 (5.9)

No significant difference on frequency of diarrheagenic *E. coli* pathotypes was found between cases and controls (*p* > 0.05)

## Discussion

The contribution of EIEC, DAEC and EHEC on the burden of diarrheal diseases is currently unclear in most sub-Saharan countries, including Mozambique. This study, provides insight, to the importance of less studied pathotypes of DEC in rural context. Our data suggest that, despite the lower prevalence of EIEC, DAEC and EHEC in Manhiça community, the presence of those pathogens in asymptomatic children is of concern, considering that this group can play significant role as carriers and prone the outbreaks for susceptible individuals.

The finding of lower prevalence of EHEC, EIEC and DAEC between November 3, 2011 and November 2, 2012 can be explained by the majority of the cases enrolled in that period were LSD; in addition that many studies have associated EHEC and EIEC to outbreaks rather than endemic diarrhea [25–28]. Interestingly, we found a variation of EHEC, EIEC and DAEC frequencies between December 10, 2007 and October 31, 2011 which only enrolled cases of MSD and matched controls. In addition, we observed similar proportion of EIEC between cases and controls and a higher frequency of DAEC in controls than in cases, suggesting the role of asymptomatic children as carriers of these pathotypes in Manhiça community. Nevertheless, phenotyping and genotyping characterization of the isolates from cases and controls could provide detailed information about their genetic relatedness.

Previous reports on DEC prevalence in Maputo city (80 km from Manhiça) described a variable circulation of EIEC (0.3–21%) in children with diarrhea [11, 14, 15], although not associated with the disease, whereas the detection of DAEC by phenotypic assay was significantly associated with diarrhea (22.8% in cases vs. 11% in controls, *p* < 0.0001) [11]. It is unknow which factors are implicated in the variation of epidemiology of EIEC and DAEC between Manhiça (rural setting) and Maputo (urban setting). However, the difference found in the frequency of DAEC between the two studies may be explained by different detection methods used (PCR vs. phenotypic assay). DAEC was classically defined by the presence of a characteristic diffuse adherence (DA) pattern on HeLa and HEp-2 epithelial cells [7, 29]. However, as EPEC and other *E. coli* can also produce a similar result to DAEC in phenotypic assay (DA pattern) [29–31], these cell adhesion assays are being considered unsuitable for identification of DAEC [32]. Furthermore, the lower prevalence of DAEC can also be explained by the molecular detection of *daaE* (less conserved gene)*,* which should be replaced by detection of recently described and highly conserved targets, such as *afaC* or *daaD* genes [32]. While some reports have associated DAEC with diarrhea [11, 33, 34], another ones have appointed for similar proportions of DAEC in case and controls [9] in alignment with our findings. In contrast with our findings, recent studies conducted in sub-Saharan Africa associated EIEC to diarrhea [35, 36]. At least, the lower circulation of EHEC is not surprisingly, since previous studies conducted in Mozambique revealed low circulation of this pathotype in Manhiça [5] and absence in Maputo city [11, 14, 15]. Nevertheless, the circulation of EHEC have been reported from a number of other locations in Africa, including Nigeria [37], and Uganda [38].

## Conclusions

This study provides a first time insight into the prevalence of DEC, particular EIEC, DAEC, and EHEC in Manhiça district. Even though, previous studies have indicated that these pathotypes are less common in endemic childhood diarrhea, special attention should be given to their transmission dynamics (e.g. the role on sporadic or epidemic diarrhea) considering that the role of asymptomatic individuals as source of dissemination remains unknown.

## Data Availability

The datasets used and/or analyzed during the current study are available from the corresponding author on reasonable request.

## Abbreviations

*E. coli*: *Escherichia coli*
DEC: Diarrheagenic *Escherichia coli*
EHEC: Enterohemorrhagic *Escherichia coli*
EIEC: Enteroinvasive *Escherichia coli*
DAEC: Diffusely adherent *Escherichia coli*
ETEC: Enterotoxigenic *Escherichia coli*
EPEC: Enteropathogenic *Escherichia coli*
EAEC: Enteroaggregative *Escherichia coli*
LSD: Less-severe diarrhea
MSD: Moderate-to-severe diarrhea
GEMS: The Global Enteric Multicenter Study
CISM: Manhiça Health Research Centre (Centro de Investigação em Saúde de Manhiça)
SHCs: Sentinel health centers
MAC: MacConkey (MAC) agar
PCR: Polymerase Chain Reaction
DA: Diffuse Adherence
